# Expediting the formulation development process with the aid of automated dissolution in analytical research and development

**DOI:** 10.1155/S1463924601000219

**Published:** 2001

**Authors:** Jon P. Sadowitz

**Affiliations:** Analytical Research and Development Barr Laboratories Inc. Pomona NY USA

## Abstract

The development of drugs in the generic pharmaceutical industry is a highly competitive arena of companies vying for few drug products that are coming off patent. Companies that have been successful in this arena are those that have met or surpassed the critical timeline associated with trial formulation development, analytical method development, and submission batch manufacturing and testing. Barr Laboratories Inc., has been successful in the generic pharmaceutical industry for several reasons, one of which includes automation. The analytical research and development at Barr has employed the use of automated dissolution early in the lifecycle of a potential product. This approach has dramatically reduced the ‘time to market’ on average for a number of products. The key to this approach is the network infrastructure of the formulation and analytical research and development departments. At Barr, the cooperative ability to work and communicate together has driven the departments to streamline and matrix their work efforts and optimize resources and time. The discussion will reference how Barr has been successful with automation and gives a case study of products that have moved with rapid pace through the development cycle


Journal of Automated Methods & Management in Chemistry, Vol. 23, No. 6 (November–December 2001) pp. 173–177

### Expediting the formulation development process with the aid of automated dissolution in analytical research and development†

**Jon P. Sadowitz**

*Analytical Research and Development, Barr Laboratories, Inc., Pomona, NY, USA*

*The development of drugs in the generic pharmaceutical industry is a highly competitive arena of companies vying for few drug products that are coming off patent. Companies that have been successful in this arena are those that have met or surpassed the critical timeline associated with trial formulation development, analytical method development, and submission batch manufacturing and testing. Barr Laboratories Inc., has been successful in the generic pharmaceutical industry for several reasons, one of which includes automation. The analytical research and development at Barr has employed the use of automated dissolution early in the lifecycle of a potential product. This approach has dramatically reduced the 'time to market' on average for a number of products. The key to this approach is the network infrastructure of the formulation and analytical research and development departments. At Barr, the cooperative ability to work and communicate together has driven the departments to streamline and matrix their work efforts and optimize resources and time. The discussion will reference how Barr has been successful with automation and gives a case study of products that have moved with rapid pace through the development cycle.*

### Introduction

Barr Laboratories, Inc., has been a competitive player in the manufacture of generic pharmaceuticals as well as proprietary products. One of the pillars of success has been the cooperative effort of the product development and analytical development teams. In the generic realm, companies who have been successful in the marketplace are those who have seized an opportunity for a viable product candidate, and been able quickly to expedite the development of that product and submit for approval. In the race for generic pharmaceuticals, there is no second place. Companies who come in second risk low market share for their product and, therefore, very little profit margin, if any.

The critical success factor becomes the speed in which a biobatch formulation can be manufactured, the analytical method developed and validated, and the biobatch or clinical batch tested and submitted to the agency.

† This paper was initially presented at the ISLAR 2000 Conference and is reproduced here by kind permission of Zymark Corporation.

### Product lifecycle

To gain a better understanding of where gains in the development and validation process can be made, one must first understand the product lifecycle time line (figure 1). First, new drug candidates are selected based on market volume, need for specialty manufacturing facilities and or techniques (i.e. narrow therapeutic range drug and others), and complementing existing product lines. Next, the candidates are selected and the products kicked off with a launch meeting which provides information about the therapy for the new product, establishing development time frames, identifying issues with development and validation, resolving issues with raw material and excipient availability, and confirming a clinical research organization for the biostudy or clinical study. At this point the evaluation/development work on the brand product begins, as well as the initial formulations from the product development side.

Once trials have been tested, preliminary decisions are made as to the direction for the analytical methodology. Automation usually enters the picture at some point close to the final formulation. Once the final formulation is reached, it is scaled up and the analytical validation proceeds along with the validation of the automated methodology. At just about the same time, both the manual and automated methods are completed and updated as the established methods for testing and releasing the product. The biobatch is made and the process validation tested by the established methods. The stability study follows at the predetermined time-points, the product is submitted to the FDA, and the methods are transferred to the quality control department.

### Statistical design

The product development team utilizes several tools to enhance its ability to provide a formulation for testing. One is the use of mathematical models for formulation design. The basic goal in utilizing statistical design is to optimize a formulation. The most readily used design schemes are the factorial design and the simplex design. These design schemes allow the formulation scientist to vary certain independent parameters while monitoring other dependant parameters.

The simplex lattice design is a relatively new design in pharmaceutical research but is quite effective (figure 2). The basis of this design scheme is that there is a constraint on the mixtures of components. The total amount

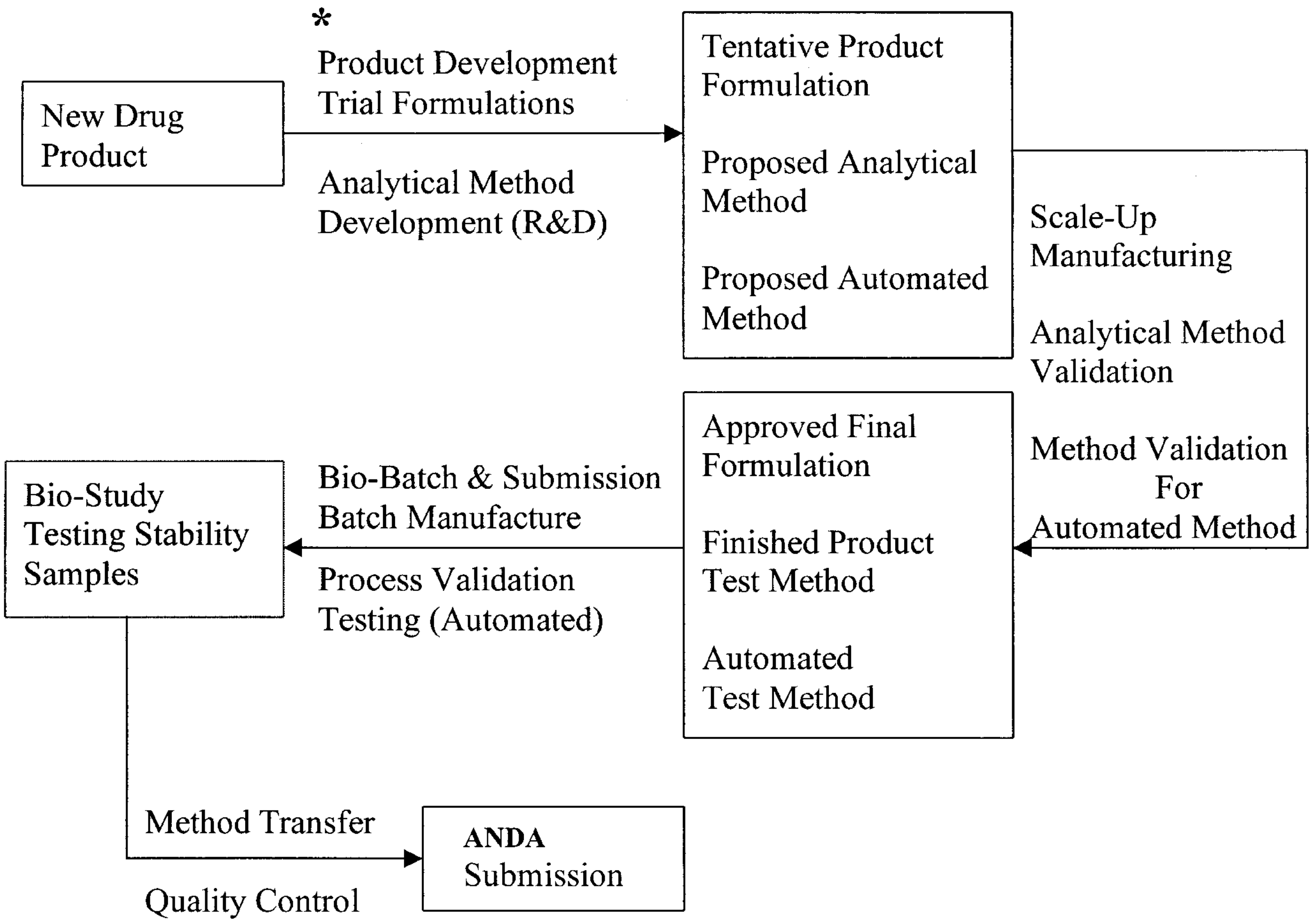

*Figure 1. Product lifecycle time line.*

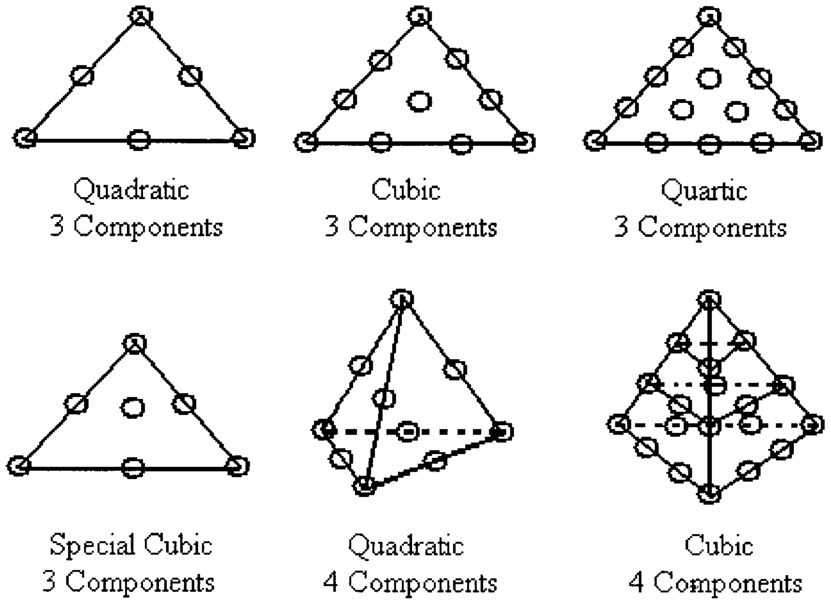

*Figure 2. Lattice structures.*

of the varying ingredients under evaluation must also sum to unity. The components of the design are then arranged in one of several lattice configurations based on the model for that product. Mathematically, the model is represented by polynomials. Using the polynomials; equations, predictive information about the model can be achieved in a short period with a limited number of test cases.

The lattice design can easily be represented by simple graphical constructs. For mixtures containing three components, a triangle can be used, and for four components, a tetrahedron. With more than four components it becomes increasingly difficult to represent a fourth dimension and higher visually, but it can be represented by an $n$-sided figure in an $(n - 1)$ dimensional space. However, the ability to uncover information about the blend of components comes by investigating the entire ranges of the composition, which is represented by the ordered arrangements of the lattice structure. The responses or features are measured at the lattice points and equations representing those reponses are derived. Calculations are performed and resultant statistics give predictive information as to the suitability of the model for that product.

Overall, these design schemes make it possible to choose a small number of informative test cases that will allow the formulation scientist quickly to resolve a formulation with the desired properties. With these abilities, the formulation scientists can make several lots of product initially and submit them to the analytical laboratory for testing. The information gained from these trials allows them to make an informed decision as to the direction of their formulation work. At this point, the samples are submitted to the analytical laboratory for analysis.

### Automation approach

The Analytical Research and Development (AR&D) laboratory at Barr handles all method development and testing of trial batches produced by the product development (PD) formulation scientists. Up until a restructuring of AR&D, all method development and validation of automated methods did not start until the beginning of the scale-up manufacturing phase (figure 1).

This approach had significant gains in the management of work flow for the individual teams whose project was being automated. In many cases, the scale-up testing and biobatch were tested completely using automation. This allowed other members of the team to concentrate on more difficult and time-consuming tasks. As a productivity tool, automation had scored A's. However, in the category of increasing the number of submissions to the agency, automation was scoring very low.

It was not until the management of the AR&D looked at how and where automation was being implemented that there would be changes in the way in which automation would be utilized to increase the number of submissions. That change in perspective caused a shift in the way in which projects would be evaluated and distributed among the different AR&D teams. The new view was to hand-pick projects at launch meeting time that would be good candidates for automation. Then, from the start of the product development trial formulation phase, all work submitted to the AR&D laboratory for dissolution or assay and CU testing would be tested using automated means.

The first product to use this approach was launched in record time for Barr. The time from launch meeting to submission was 6 months (including the 3-month stability time-point). Each additional product that has utilized this approach has been expedited as a result of the quick sample turn around. Why was Barr so successful in this approach? The answer in the infrastructure of the company and the successful experience of veteran automation users provided with the right tools.

### Infrastructure

The infrastructure of Barr is such that a large number of projects can be handled by the individual development teams (figure 3). In turn, these development teams correspondingly work with the AR&D teams for developmental testing of their formulations. Not only does this approach lend itself well to highly parallel development of products, but the skill sets of each group get developed and most optimally utilized within their respective environment.

The workflow (figure 4) comes as a function of the PD's submission of samples to the AR&D laboratory for analysis. By changing the entrance of automation to the beginning of the project, trials could be evaluated utilizing automated systems. PD's approach to submitting samples has changed as well. The open lines of communication, matrix design schemes and the high-volume capacity of automation has caused an acceleration in the development timeline for products. Utilization of automation has increased the volume of information

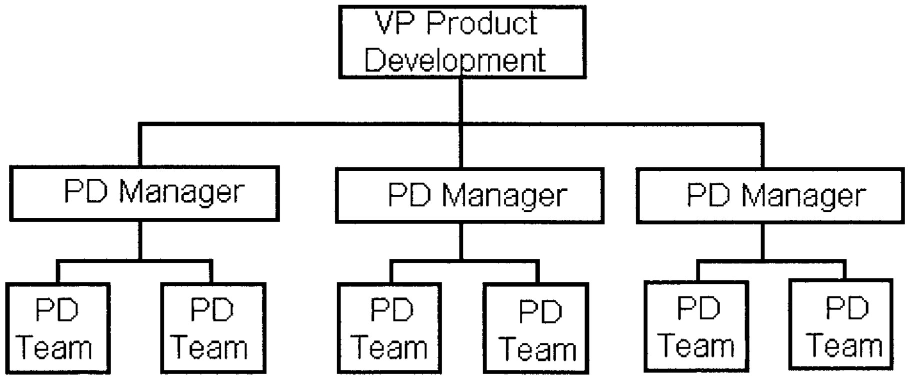

*Figure 3. Infrastructure at Barr.*

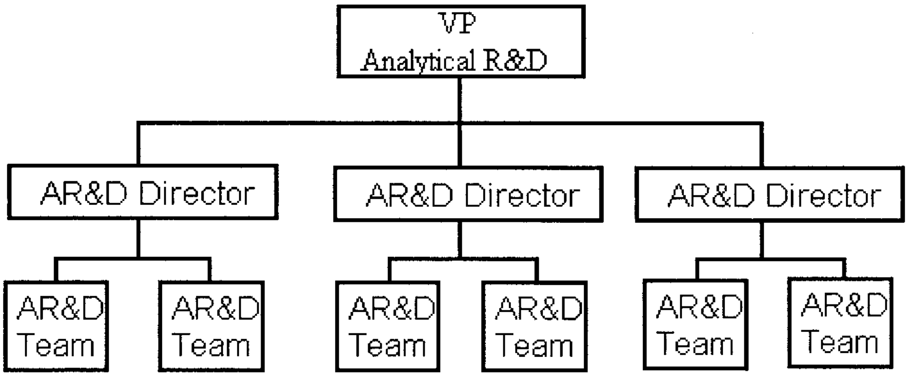

*Figure 4. Workflow at Barr.*

while decreasing the overall time for the development of the product.

**Automation tools**

Analytical R&D has made use of several automation resources in its efforts to push the envelope of the critical timeline associated with launching new generic and proprietary products. One of the most useful tools has been the automated dissolution workstation (figure 5). We have employed the use of Zymark's Multidose Automated Dissolution Workstation (MADW) as the centrepoint of Barr's streamlining approach at launching products.

Some of the key features of the multidose that make it suited for high throughput analysis are its ability to run up to eight batches without reloading, to utilize four different media and DI $H_2O$, to run either apparatus I or II to collect samples in vials or test tubes, or to run utilizing on-line UV analysis.

The coordination of the interdepartmental communication is the most important factor that has allowed all of the pieces to work together to optimize turnaround time. Our approach in this area has been to set-up the development project with the PD department in such a way that high-volume testing is part of the resource for testing samples. With this in mind, the formulation scientists have utilized the different statistical design models to submit groups of samples to be analyzed with several different media. The batch analysis approach has shown the effectiveness of the automation tools by providing large volumes of development information in a relatively short period.

**Efficiencies**

The old approach in development was to submit a sample or two to the laboratory for analysis. The next day, results would be generated and submitted for review by the formulation scientists. Upon their review, decisions as to the results would be made and another one or two samples would be submitted to the laboratory for analysis on the following day. After 1 week, only about six trials would have been made and tested.

In the new approach, a set of samples of beween four and eight would be submitted for analysis in media ranging from 0.1 N HCl through the pH range to pH 8.0 buffer. The results of these analysis would generally be submitted by 10:00 hours the next day and we could expect to receive more trial samples to analyse by late that day. In effect, the company could formulate, analyse, calculate and review, and reformulate within 24 h.

As a result, the projects have begun to move rapidly through the development and validation stage. These changes have also affected the other areas of the company causing them to streamline their efforts to keep pace with

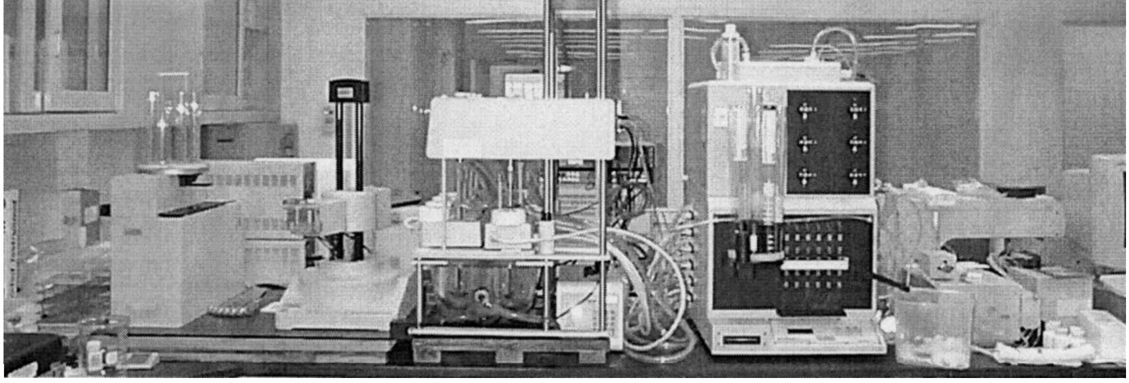

*Figure 5. Automatch dissolution workstation.*

the fast-moving projects. Use of data analysis tools has also increased steadily as the information technology (IT) department has implemented systems to improve data analysis and report generation.

### Case studies

Currently, there are several projects that have benefited from the use of automated dissolution. The products themselves range in the several categories of therapy. In each case, the approach was the same: to implement the use of automated dissolution from the project inception to the completion of the submission.

Three of the products were developed using on-line UV analysis. The other product was off-line LC collection into vials. For the products that used on-line UV analysis, there was an added benefit of additional time-point analysis that compromised no efficiency in the throughput. It was not more difficult to analyse the dissolution at 5-min intervals than at 30-min intervals. This allowed for a greater number of sampling points to provide the formulation scientists with more data about the dissolution analysis. The initial dissolution work for all product was conducted by running a series of dissolution analysis of the brand product by both basket and paddle in several media representing the pH of the gastrointestinal tract. This information coupled with differing RPM and peak vessel combinations provided a wide spectrum of detailed information about the nature of the brand product. For the example mentioned above, the development time for Barr's formulation was just under 6 weeks. This compressed timeline was achieved because of the volume of data generated early in the life of the project. A final formulation was achieved and confirmed, and a series of additional dissolution tests in different media conducted to confirm the desired profile. Shortly thereafter, a scale-up formulation was manufactured and tested. All of the analytical methods (both manual and automated) were validated and all samples for the biobatch were analysed using the automated methodology. The project from start to submission, including the 3-month stability time-point interval, was 6 months. Compare that with the usual 9–18 month timeline for product development and submission.

To be realistic, not all products can follow such an aggressive pace. There are raw material and excipient availability problems that need to be contended with as well as issues that arise from manufacturing and analytical areas. Two of the product currently in the pipeline for automation have experienced these problems and have slowed the pace somewhat, but in the grand scheme of projects, that has allowed an opportunity to evaluate and develop other products in the interim.

The remaining product has been placed on a fast track and has been developed to date in a record time: 3 weeks. This project is far from complete, but final formulation will be soon to arrive and this project should be submitted by early 2001. That would be a 5-months development and submission timetrame.

### Conclusion

The use of automation in the analytical research and develoment department has been a major factor in expediting the formulation development process. However, it is not the sole factor for the success. Barr Laboratories, Inc., success at expediting product development is attributed to its corporate commitment to bringing healthcare products to the market quickly. The cooperation of the differing departments in working toward the goal of accelerated development has been the key to making the process successful.

Barr continues to look at new technology and new approaches to improving and accelerating product development and will continue to invest in those areas that will allow it to do so.

### Acknowledgements

The author thanks the following for their contributions. Dr Emad Alkhawam for his commitment to supporting and developing automation in analytical research and development. Product Development for the contribution of product development information and the AR&D Robotics Team for their dedicated efforts in support of product development trial batch, scale-up and Biobatch testing.

